# Effect of sulfonation degree on molecular weight, thermal stability, and proton conductivity of poly(arylene ether sulfone)s membrane

**DOI:** 10.1080/15685551.2016.1231035

**Published:** 2016-09-21

**Authors:** Majid Pirali-Hamedani, Shahram Mehdipour-Ataei

**Affiliations:** ^a^ Polyurethane and Advanced Polymeric Materials Department, Iran Polymer and Petrochemical Institute, Tehran, Iran

**Keywords:** Poly(arylene ether sulfone), fuel cell membrane, synthesis, thermal stability, structure-property relations

## Abstract

Direct copolymerization of sulfonated and non-sulfonated difluorodiphenyl sulfones as dihalide monomers with hydroquinone and also 4,4′-(4,4′-sulfonylbis-(1,4-phenylene)bis(oxy)) diphenol as diols led to preparation of two series of poly(arylene ether sulfone)s. Copolymers with different degrees of sulfonation (40, 50 and 60%) were synthesized in order to evaluate their potential for fuel cell application. ^1^H-NMR, FT-IR, and mass spectroscopy were used for characterization of prepared monomers and copolymers. Differential scanning calorimetry and thermogravimetric analysis were applied for investigation and comparison of the thermal properties of copolymers. Laser light scattering (LLS) was employed to calculate zeta potential, conductivity, and molecular weight of copolymers. Copolymers were obtained in high and sufficient molecular weight that was basic need to reach reasonable physical and thermal properties for applications as fuel cell membrane. The effect of similar structural repeating units with different sizes on the final properties of sulfonated poly(ether sulfone)s was investigated to compare their potential in fuel cell membrane.

## Introduction

Nowadays, polymer scientists have been focused on the novel and market-oriented applications of polymers due to their wide variety of chemical compositions, availability, processability, and anticipated stability for the vast applications.[1–6] Among different polymers, preparation of derivatives of poly(arylene ether) polymers such as poly(arylene ether ether ketone) (PEEK), poly(arylene ether sulfone) (PAES) and their relatives has been widely reported and being the focal point of many researches due to their unique and specific properties.[7–9] Poly(arylene ether sulfone)s are known as a class of high-performance thermoplastic engineering polymers with remarkable properties including excellent resistance to hydrolysis even in the presence of acids and bases, well mechanical and thermal properties, and excellent resistance to oxidative degradation.[10–12] Based on these specifications, they are used in a variety of applications including coating, ultra-filtration, biomaterial, adhesives, and fuel cell.[13–15] There are still further demands to modify the structure of PAESs to obtain more favorable features for extending their applications. The best approach to modify PAESs for application as a proton exchange membrane (PEM) is to employ electrophilic aromatic sulfonation on aromatic rings.[16] There are two ways to sulfonate these polymers, the first one is sulfonation of polymer main chain after polymerization (post-sulfonation), and the second one is monomer sulfonation before polymerization step.[17] The main difference between these two methods is control of the content and position of sulfonic acid groups in the main chain.

The first pioneering report in the field of post sulfonated PAESs was from Noshay and Robeson [18] by introducing a procedure for the bisphenol A-based poly(ether sulfone). Different sulfonating agents have been reported for this polymer modification.[19–21]

In the post-sulfonation method, the position of sulfonic acid groups is on the electron rich rings of polymer chain that are next to ether groups. In this method, the position and concentration of sulfonic acid groups could not be controlled. Also, when aromatic groups of polymer chain are so active, post-sulfonation might lead to crosslinked polymers. In sum, post-sulfonation method has many disadvantages such as polymer decomposition and side reactions that decrease thermal and mechanical properties of the samples.[22,23]

In the second method (monomer sulfonation) the position, concentration, and distribution of sulfonic acid groups could be simply and completely under control. Also, the probability of crosslinking by reaction path tends to zero. In opposite of post-sulfonation, in this method introduction of two sulfonic acid groups to each repeating unit of PAESs is possible.

Sulfonated monomer for flame retarding materials was prepared by Robeson and Matzner [24] the pioneering scientists in this field. Recently, Ueda et al. [25,26] reported the sulfonation of 4,4′-dichlorodiphenyl sulfone and other researchers [27–34] modified this procedure for the disulfonation of monomer.

Generally, sulfonated poly(ether sulfone)s (SPES) could be prepared via reaction of sulfonated and non-sulfonated dihalide monomers with different types of aromatic diols. Savariar and his coworkers [35] synthesized poly(biphenyl ether sulfone) based on the reaction of 4,4′-dichlorodiphenyl sulfone with biphenol, also Zutty [36] reported reaction of 4,4′-dichlorodiphenyl sulfone with 4,4′-dihydroxydiphenyl sulfone for preparation of PES. Robello [37] synthesized moderate molecular weight, highly crystalline, and extremely high and unusual thermally stable polymer by self-condensation of 4-fluorobenzenesulfinate. Yokozawa [38] prepared poly(ether sulfone)s with defined molecular weight and low polydispersity via polycondensation of 4-fluoro-4′-hydroxydiphenyl sulfone in the presence of (fluorophenyl) (trifluorophenyl) sulfone as initiator and 18-crown-6 in a chain-polymerization manner in sulfolane. Kricheldorf et al. [39] prepared poly(ether sulfone)s by polycondensation of silylated 4-tert-butylcatechol with 4,4′-difluorodiphenylsulfone in N-methylpyrrolidone. They studied the influence of stoichiometry and conversion on the molecular weight and extent of reaction through the effect of feed ratio and the reaction time. Weisse [40] reported copolymerization of 5-[(4-fluorophenyl)sulfonyl]-2-fluorobenzoic acid with bis(4-hydroxyphenyl)sulfone in 11-dioxothiolane with sodium carbonate as a base to obtain carboxylated poly(ether sulfone)s. Wang [41] prepared novel partially fluorinated poly(arylene ether sulfone)s with pendant quaternary ammonium groups by copolymerization of 2,2′-dimethylaminemethylene-4,4′-biphenol, and 4,4′-biphenol with 3,3′,4,4′-tetrafluorodiphenylsulfone. The resulted copolymers showed high molecular weight and outstanding solubility in polar aprotic solvents. Polymerization of fluorene-based poly(arylene ether sulfone) copolymers containing tetra-methoxy groups via a new bisphenol monomer, i.e. 99-bis(35-dimethoxy-4-hydroxyphenyl) fluorene has been reported.[42] Transformation of the methoxy group to the reactive hydroxyl group and the relative side-chain type sulfonated copolymers (SPAES) by sulfobutylation has been described. Their polymers showed lower water uptake and higher proton conductivity in comparison with some reported sulfonated poly(arylene ether sulfone)s containing pendent sulfophenyl groups. Changkhamchom reported composite proton exchange membranes of sulfonated poly(ether ketone ether sulfone) (S-PEKES) and molecular sieve for direct methanol fuel cell.[43] Mehdipour-Ataei prepared two groups of poly(arylene ether sulfone)s with different diols (bisphenol-P and bisphenol-M) and compared the effect of chain structure on methanol crossover in direct methanol fuel cell.[44] Nanocomposite blends of fully sulfonated poly(ether ketone)/non-sulfonated poly(ether sulfone) as PEMs from dual electrospun mats was another research of his group.[45]

In the present work, two groups of novel poly(arylene ether sulfone)s with different structures were prepared by direct polymerization of sulfonated and non-sulfonated dihalide monomers with hydroquinone and also 4,4′-(4,4′-sulfonylbis-(1,4-phenylene)bis(oxy)) diphenol (SPHD). In order to compare the effect of repeating units on the final properties of these sulfonated poly(ether sulfone)s (SPES), the chemical structure, molecular weight and thermal properties of these two series of polymers were studied. These groups of polymers revealed potential application in fuel cell membrane.

## Experimental

### Material

Hydroquinone (HQ) was purchased from Merck and was recrystallized from hot water before use. 4,4′-(4,4′-Sulfonylbis-(1,4-phenylene)bis(oxy)) diphenol was synthesized from 4,4′-difluorodiphenyl sulfone and hydroquinone. 4,4′-Difluorodiphenyl sulfone was provided from Merck and recrystallized from toluene, and 3,3′-disulfonated-4,4′-difluorodiphenyl sulfone (DFS) was synthesized from nonsulfonated dihalide and recrystallized from (water/2-propanol) mixture. Potassium carbonate (Merck) was dried at 120 °C in a vacuum oven overnight. Dimethylacetamide (DMAc) from Merck was dried over calcium hydride for 12 h and then distilled under reduced pressure and stored over molecular sieves. Ethanol, toluene, 2-propanol, fuming sulfuric acid (30%) (All from Merck) were used as received.

### Monomer synthesis

#### Synthesis of 4,4′-(4,4′-sulfonylbis-(1,4-phenylene)bis(oxy)) diphenol (SPHD)

Typical procedure was as follows: Hydroquinone (6 mmol), 4,4′-difluorodiphenyl sulfone (3 mmol) were added into a 3-necked flask equipped with nitrogen inlet and a Dean Stark trap. Potassium carbonate and 12 mL of DMAc were introduced to the mixture. Then 10 mL of toluene was added as an azeotropic agent. The reaction mixture was refluxed at 140 °C for 4 h. The temperature was raised slowly to 175 °C by controlled removal of toluene and the reaction was continued for 30 h. The solution was cooled to room temperature and the mixture was poured into deionized water and stirred overnight, then it was washed thoroughly with deionized water. Finally, the filtered monomer was vacuum dried at 120 °C for 24 h.

#### Synthesis of 3,3′-disulfonated- 4,4′-difluorodiphenyl sulfone sodium salt (SDFS)

4,4′-Difluorodiphenyl sulfone (20 mmol) was dissolved in 12 mL of 30% fuming sulfuric acid. The solution was heated and stirred in an oil bath at a temperature of 110 °C for 6 h and after cooling, the mixture was poured into the 100 mL of ice water. The solution was titrated with 6 N NaOH to neutralize the excess of sulfuric acid. Then, the solution was poured into the excess amount of ethanol and resulted white precipitate of Na_2_SO_4_ was filtered. At the next step, ethanol was removed from the filtrate by distillation. After distillation, the solution of sulfonated monomer in water was left. By pouring an excess amount of 2-propanol in the solution, white needle-like crystals of sulfonated monomer was appeared. The product was filtered and dried in a vacuum oven at 120 °C for 12 h.

### Copolymers synthesis

Direct synthesis of sulfonated copolymers was achieved by a typical procedure using nucleophilic aromatic substitution reaction.[30,44] HQ or SPHD diols (6 mmol), DFS dihalide (3.3 mmol), and SDFS (2.7 mmol) were added into a 3-necked flask equipped with nitrogen inlet and a Dean Stark trap. Potassium carbonate (6.9 mmol) and 16 mL of DMAc were put into the mixture. Then 8 mL of dry toluene was added as an azeotropic agent. The reaction mixture was refluxed at 140 °C for 4 h and gradually it was heated to 175 °C by controlled removal of toluene. Heating of the reaction was continued for 30 h to viscose polymer solution was obtained. The solution was cooled to room temperature and diluted with 16 mL of DMAc. Then, the mixture was poured into deionized water and the obtained white fibrous precipitate stirred in deionized water overnight at 50 °C to remove remained salts, then it was washed thoroughly with deionized water. Finally, the filtered copolymer was vacuum dried at 120 °C for 24 h.

### Membrane preparation

Sulfonated copolymers in potassium salt form were cast onto a glass plate from their DMAc solution (5%). Then they were subjected to suitable heating cycle for solvent removal and uniform film formation. These membranes were converted to their acid forms by immersing them in 4 M H_2_SO_4_ solution for 24 h. After that, the obtained membranes (in acid form) were immersed in deionized water for another 24 h and washed several times with deionized water. The obtained membranes were dried in a vacuum oven at 60 °C overnight. The thicknesses of all transparent and flexible membranes were in the range of 50–100 μm.

## Characterization of monomer and copolymers

### Mass, ^1^H-NMR, and FT-IR spectroscopy

Mass spectrometry of the synthesized monomers was performed on an Agilent Technology (HP) to measure their molecular weights. Nuclear magnetic resonance spectra of the monomer and copolymers were recorded for structural characterization with a Bruker Avance DPX 500 MHz. DMSO-d_6_ was used as solvent and TMS as the internal reference. Also, the molecular structure and functional groups of the monomer and copolymers in thin film samples were studied using FT-IR spectroscopy on a Bruker- IFS48 FTIR spectrometer.

### Inherent viscosity

Inherent viscosity of the polymers was measured with an Ubbelohde capillary viscometer at a concentration of 0.5 g /dL in DMAc as the solvent at 25 °C.

### Refractometer and laser light scattering

The refractive index of the synthesized copolymers and solvents was calculated with an Abbe refractometer and the data were used for laser light scattering (LLS). Laser light scattering was employed to calculate zeta potential, conductivity, and molecular weight of copolymers. LLS measurements were made on a Malvern Zetasizer instrument in which copolymers were dispersed in Dimethylacetamide (RI = 1.4375, viscosity = 0.945cP) at room temperature.

### Thermogravimetric analysis and differential scanning calorimetry

Thermogravimetric analysis (TGA) was employed to assess thermal stability of membranes with a Mettler TGA instrument under air atmosphere from room temperature to 700 °C at a heating rate of 10 °C/min. Differential scanning calorimetry (DSC) measurements were made on a Mettler DSC instrument at a heating rate of 5 °C/min under air atmosphere. Glass transition temperatures (*T*_g_) were taken as the middle point of the step transition.

### Water uptake and ion-exchange capacity

Membranes dried in vacuum oven at 60 °C overnight, then immersed in deionized water at room temperature for more one day, the films were taken out, dried and quickly weighted. The water uptake (WU, wt %) was calculated by the following equation:

$$\text{WU}=\left(\left(W_{\text{s}}-W_{\text{d}}\right)/W_{d}\right)\times 100\%$$

where *W*_s_ is the weights of the wet membranes and *W*_d_ is the weights of the dry membranes.

Ion-exchange capacity (IEC) of the membranes was measured by titration. The membranes were first converted to acid form and immersed in a 2 M NaCl solution for 24 h to exchange H^+^ ions with Na^+^ ions. Then, the exchanged H^+^ ions within the solutions were titrated with a 4 mM NaOH solution using phenolphthalein as an indicator. The IEC values were calculated according to the following equation:

$$\text{IEC}\left(\text{m}\;{\text{eqg}}^{-1}\right)=\left(M_{\text{NaOH}}\times V_{\text{NaOH}}\right)/W_{\text{d}}$$

where *M*_NaOH_ and *V*_NaOH_ stand for the molar concentration and volume (mL) of the aqueous NaOH solution used in titration and *W*_d_ (g) is the weight of dry membrane.

## Results and discussion

### Monomer characterization

**Figure 1.**
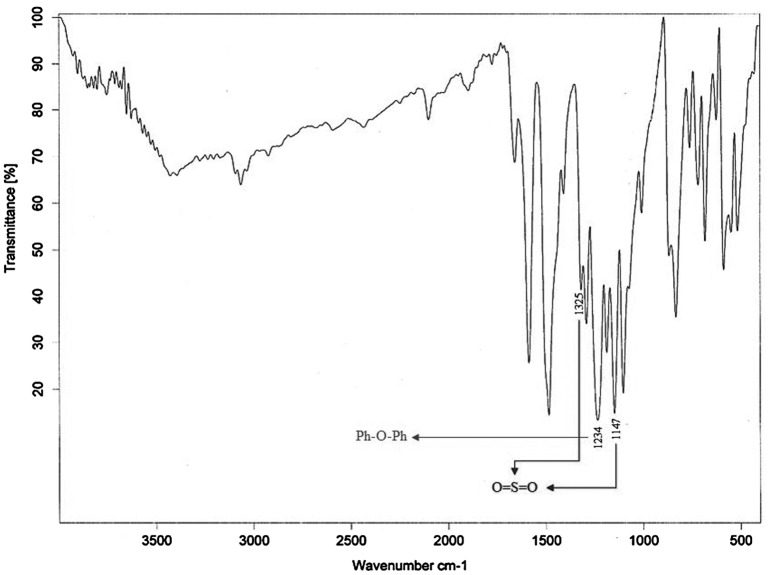
FT-IR spectrum of SPHD.

**Figure 2.**
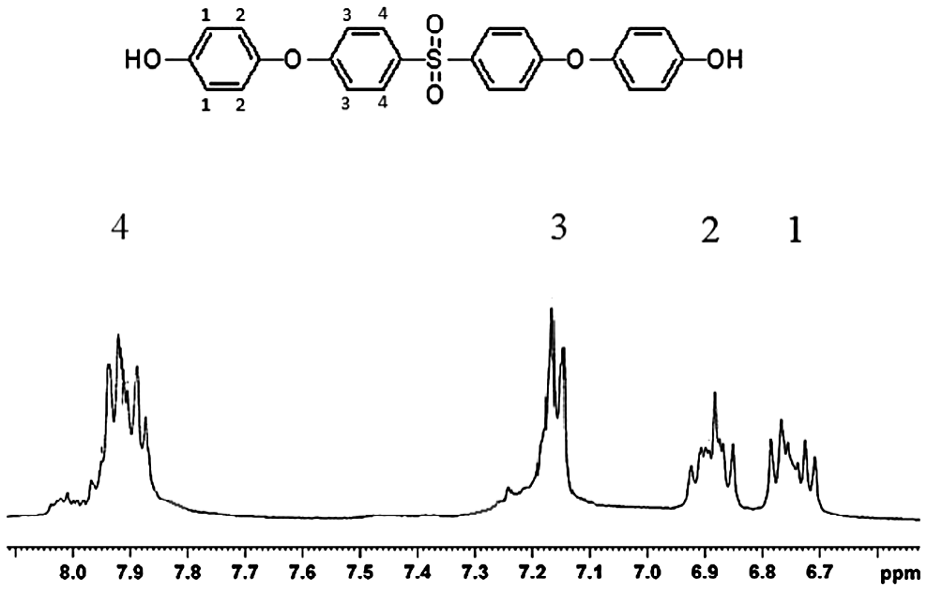
^1^H-NMR spectrum of SPHD.

**Figure 3.**
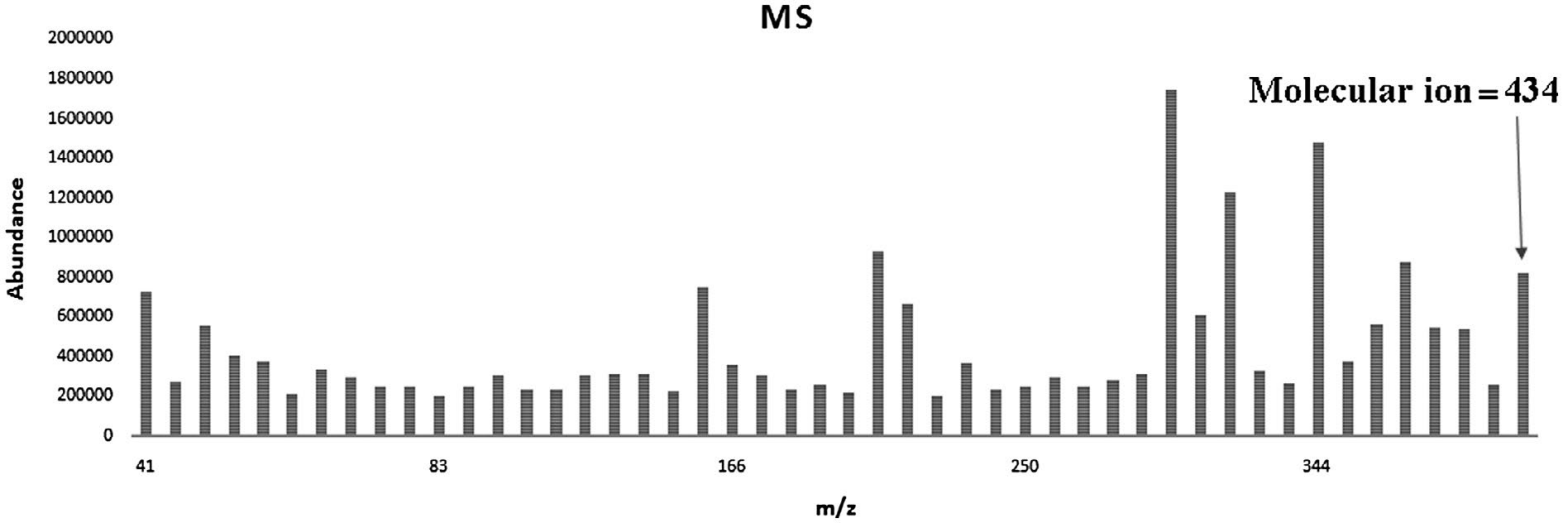
Mass spectrum of SPHD.

**Scheme 1.**
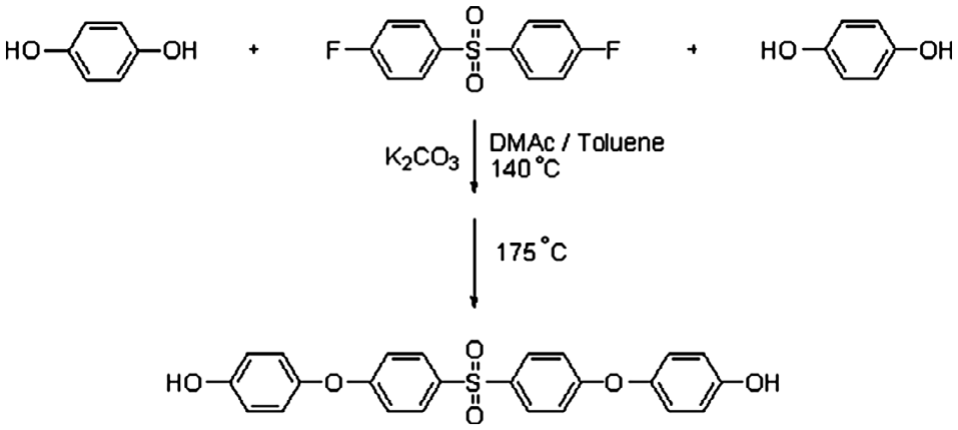
Synthesis of 4,4′-(4,4′sulfonylbis-(1,4-phenylene)bis(oxy)) diphenol.

For preparation of poly(arylene ether sulfone)s membrane, polycondensation reaction between a diol and a dihalide was considered. In this way, a new diol monomer (SPHD) was prepared via nucleophilic substitution reaction of hydroquinone with 4,4′-difluorodiphenyl sulfone in 2:1 molar ratio (Scheme 1). The chemical structure of the diol monomer was identified by FT-IR, ^1^H-NMR, and mass spectroscopy shown in Figures 1–3, respectively. In the FT-IR spectrum, the characterization bands were observed at 1147, 1325, and 1234 cm^−1^ attributed to the characteristic bands of O=S=O and Ph–O–Ph bonds, respectively (Figure 1). All the protons were assigned in the ^1^H-NMR spectrum (Figure 2) confirming the chemical structure of SPHD. Based on the mass spectrum, molecular ion of 434 was clearly observed in the pattern of spectrum, indicating formation of SPHD structure (Figure 3).

**Figure 4.**
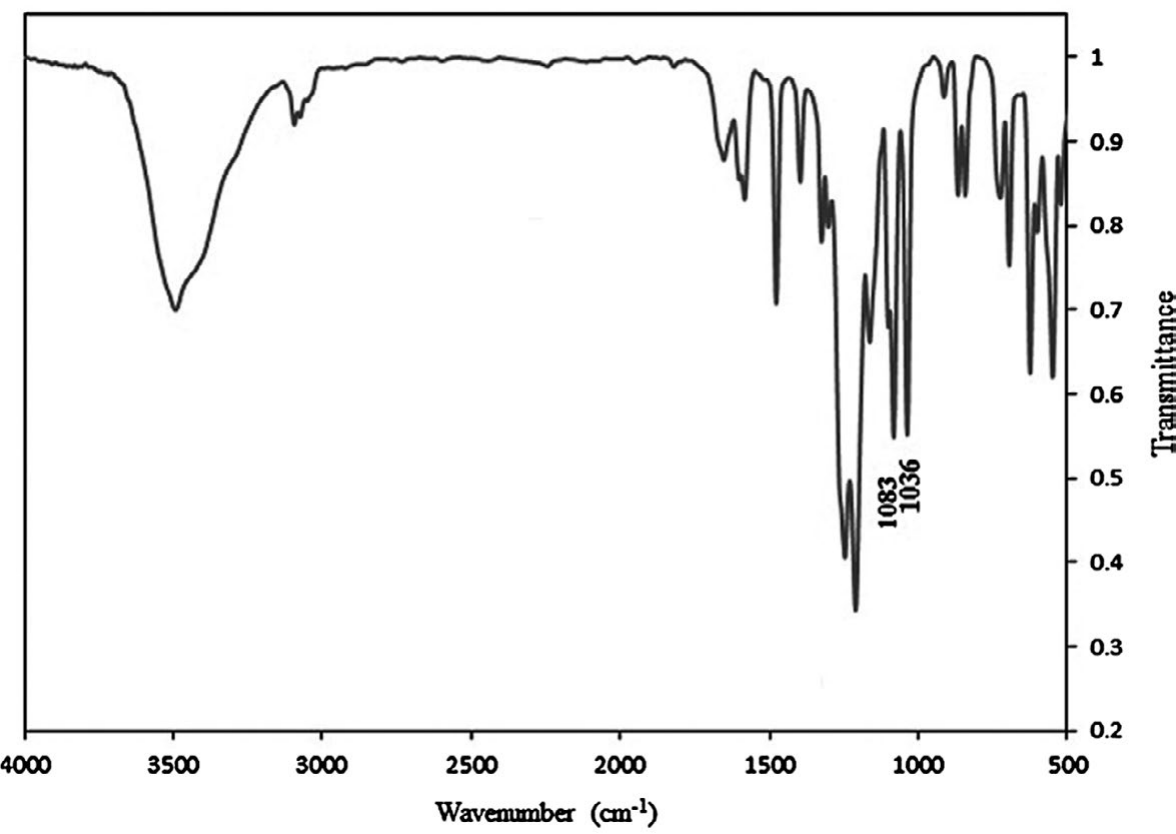
FT-IR spectrum of 3,3′-disulfonated- 4,4′-difluorodiphenyl sulfone sodium salt.

**Figure 5.**
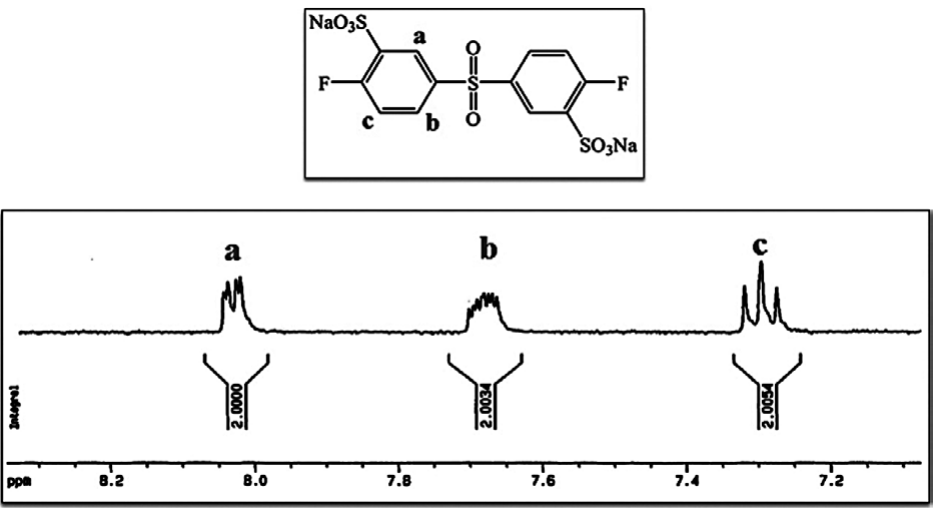
^1^H-NMR of 3,3′-disulfonated-4,4′-difluorodiphenyl sulfone sodium salt.

**Figure 6.**
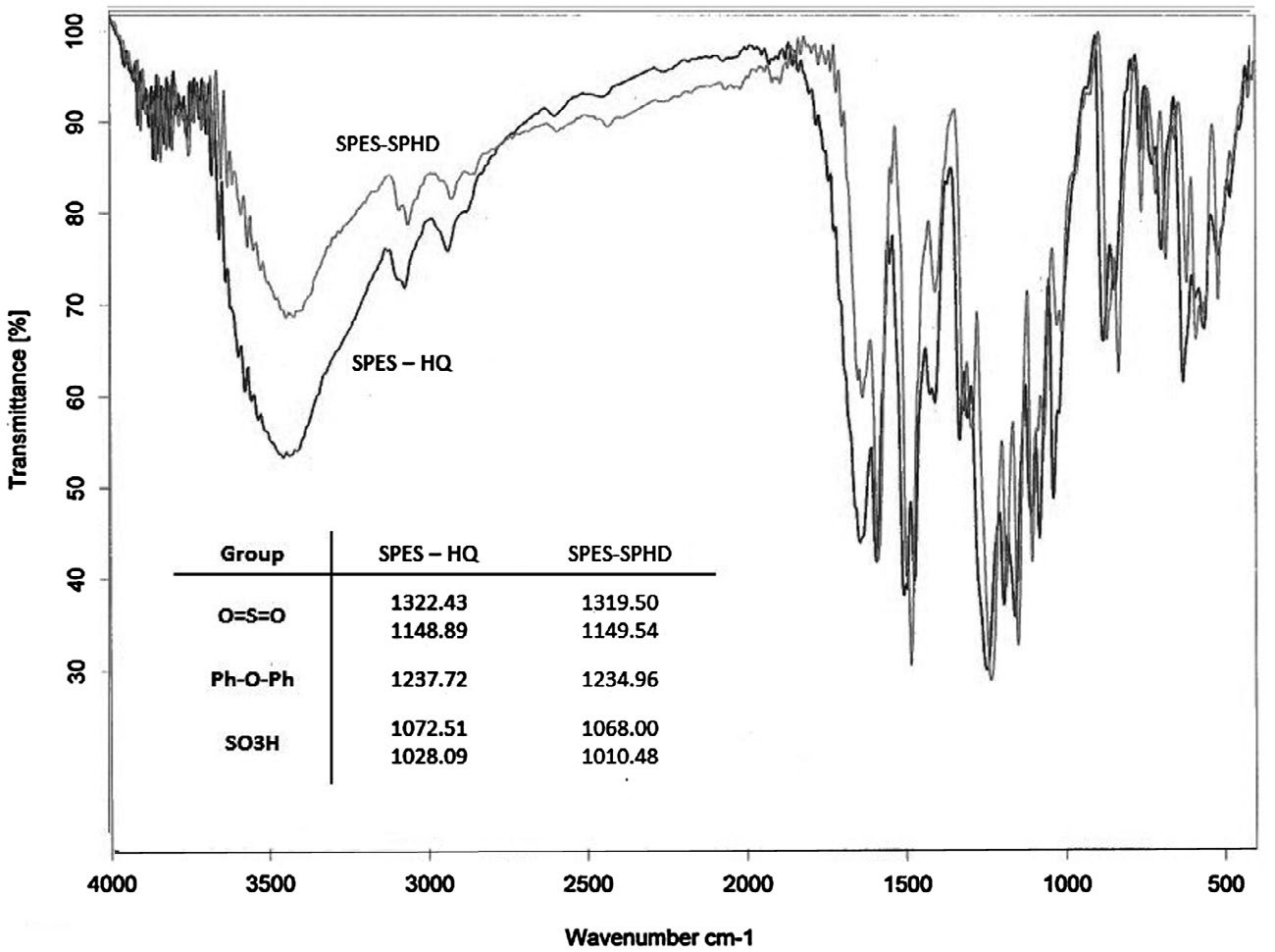
FT-IR spectrum of hydroquinone and SPHD-based poly(ether sulfone).

**Figure 7.**
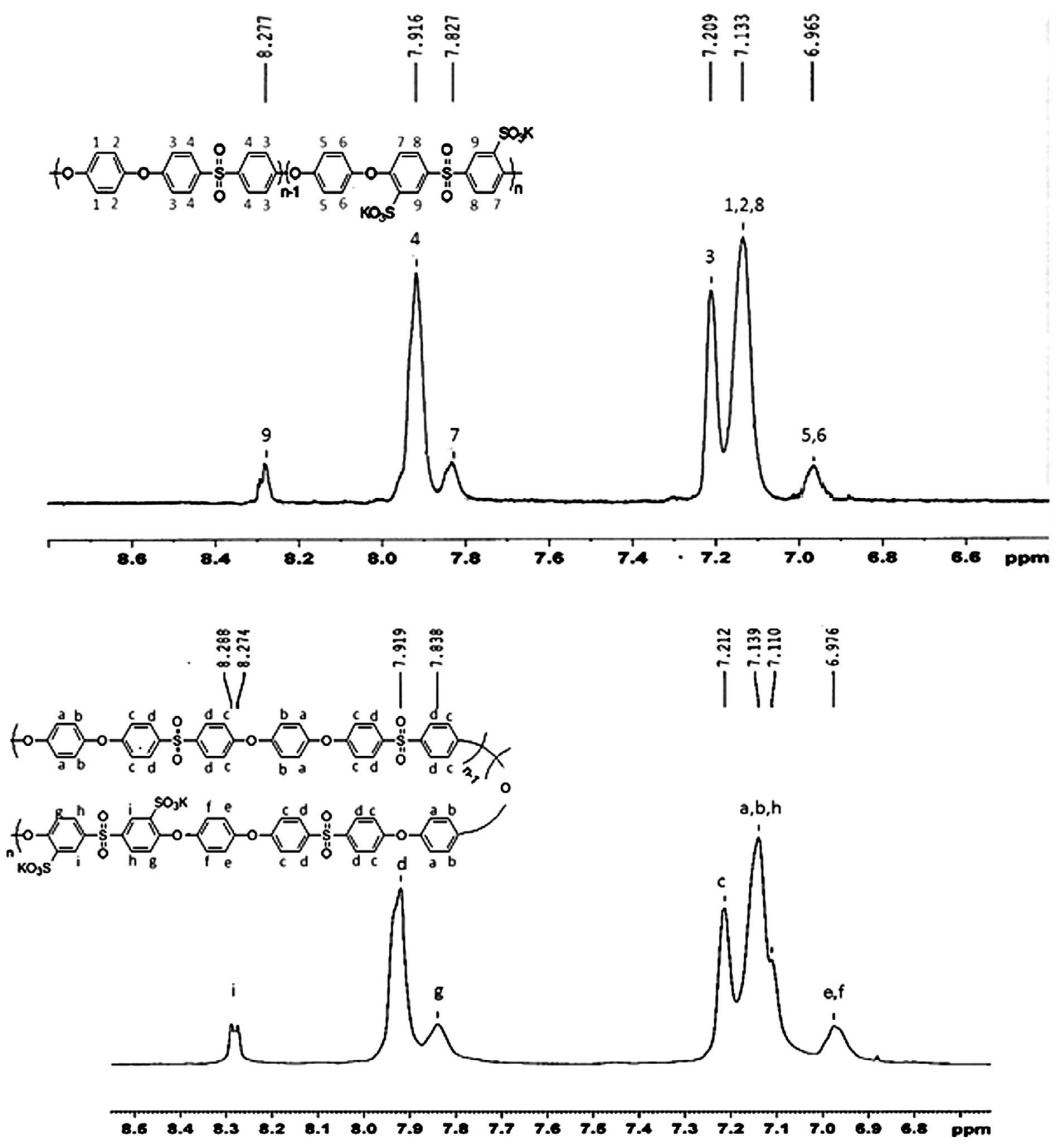
^1^H-NMR spectrum of hydroquinone and SPHD-based poly(ether sulfone).

**Figure 8.**
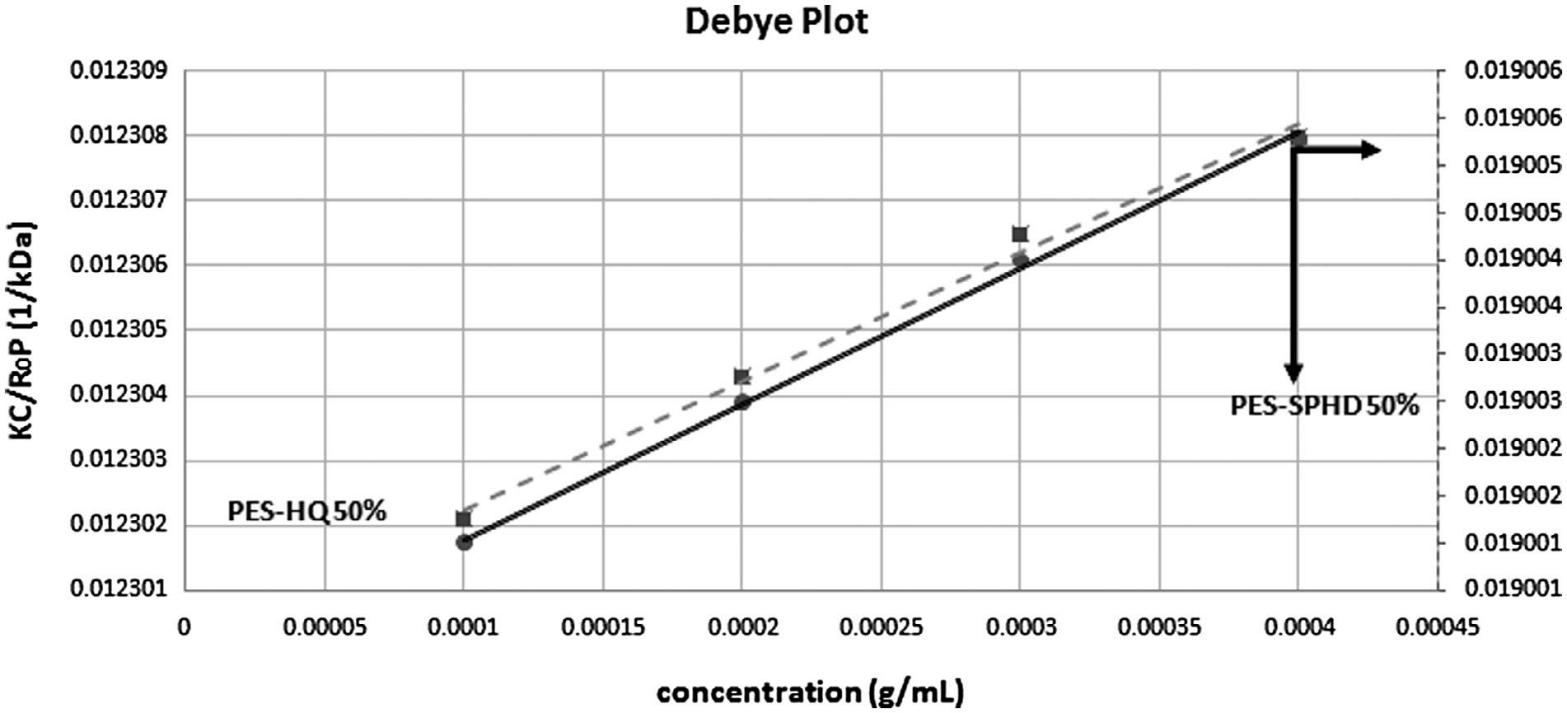
Debye plot of hydroquinone and SPHD-based poly(ether sulfone).

**Figure 9.**
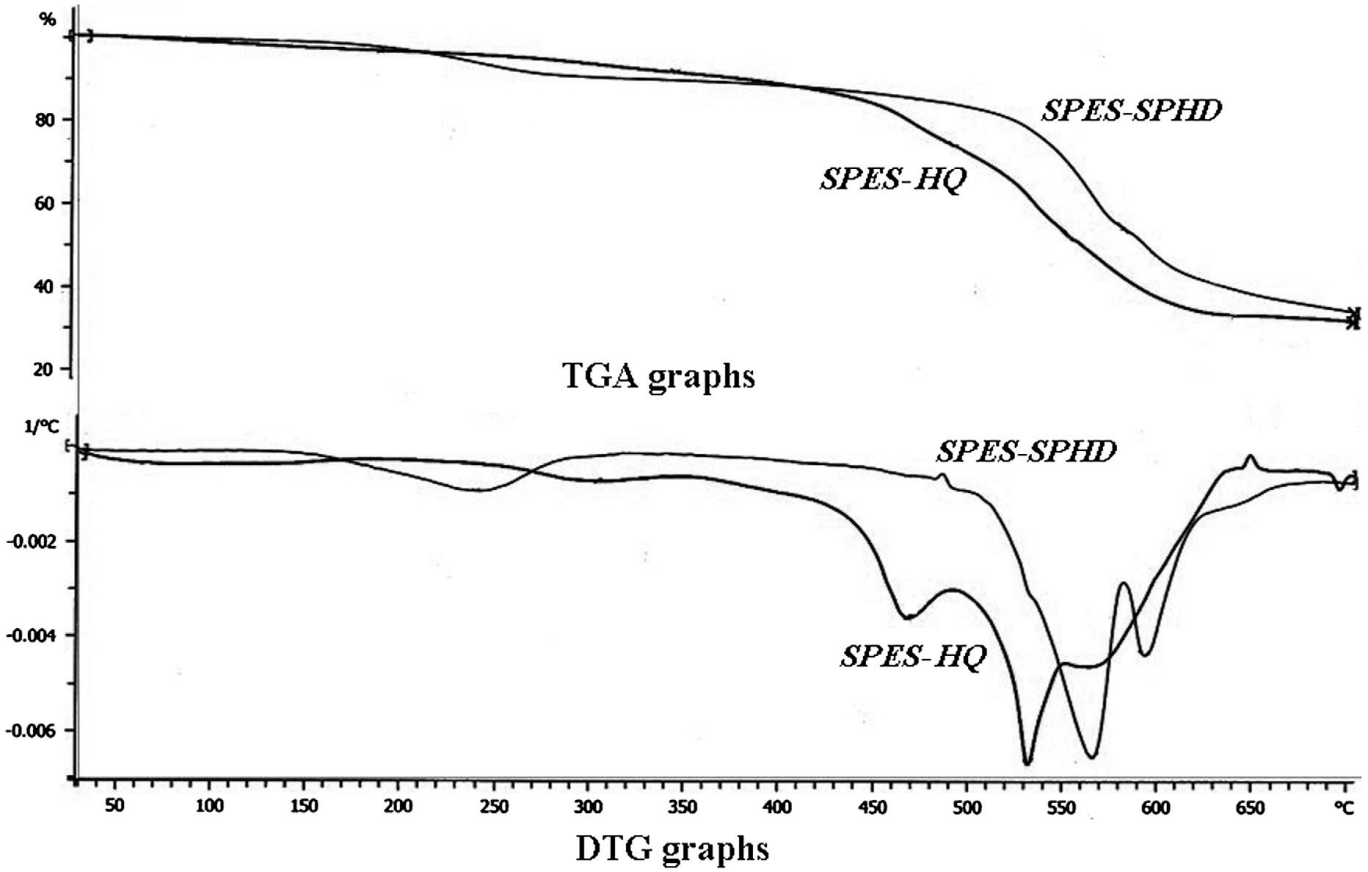
TGA graph of hydroquinone and SPHD-based PES.

**Figure 10.**
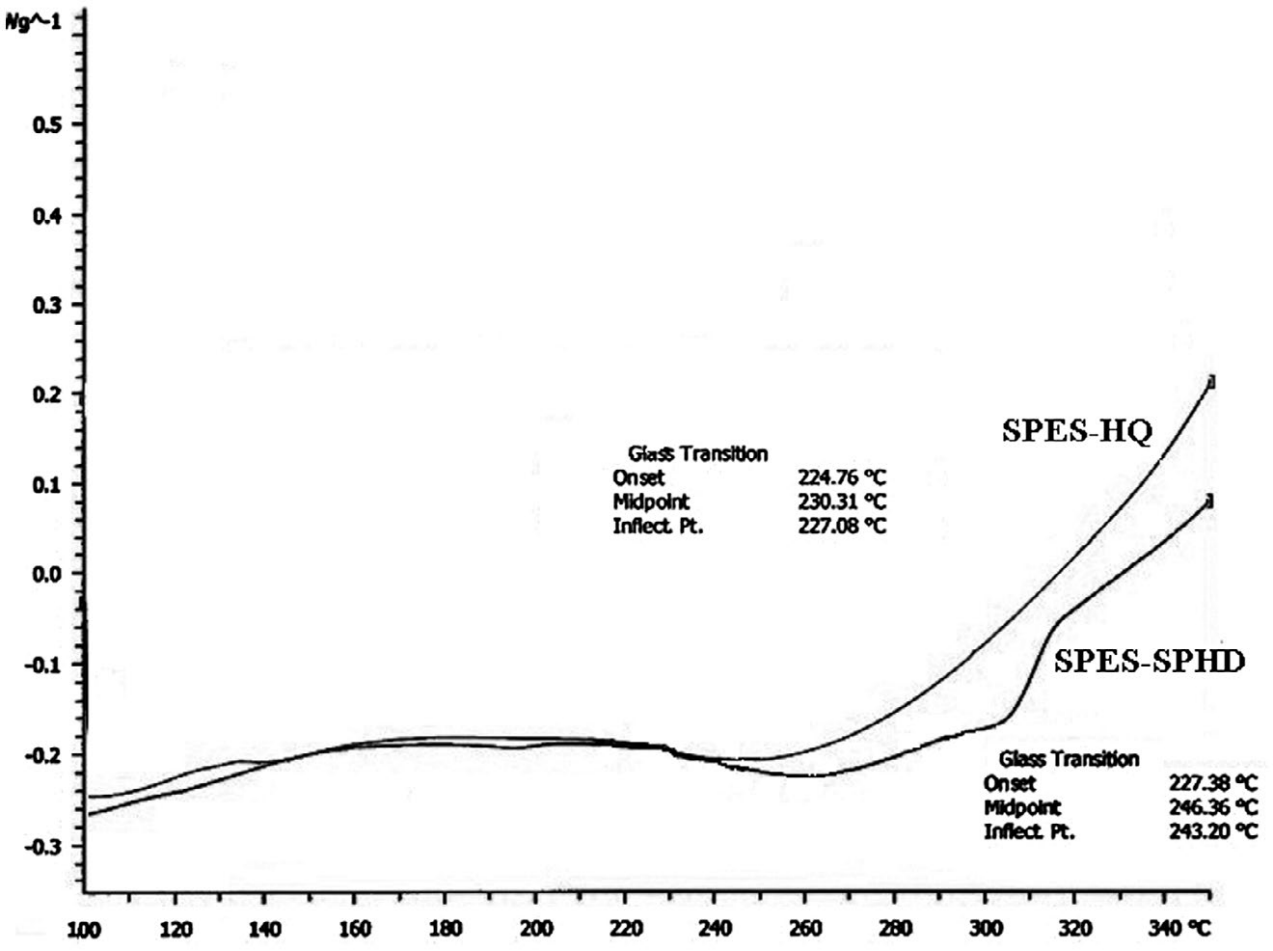
DSC graph of hydroquinone and SPHD-based PES.

**Scheme 2.**
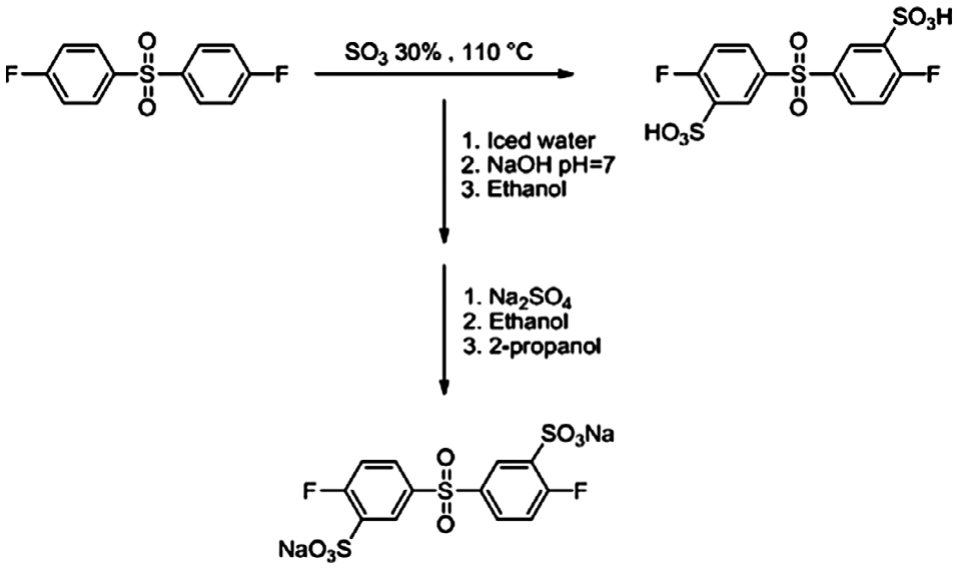
Synthesis of 3,3′-disulfonated-4,4′-difluorodiphenyl sulfone sodium salt.

3,3′-Disulfonated-4,4′-difluorodiphenyl sulfone (SDFS) was synthesized via electrophilic aromatic sulfonation of 4,4′-difluorodiphenyl sulfone using fuming sulfuric acid (Scheme 2). Since preparation of pure monomers is one of the basic parameter to obtain high molecular weight polymer, different method have been reported for purification of sulfonated dihalides in the literature. In the present work, purification procedure was achieved based on water-isopropyl alcohol method [46,47] and pure SDFS monomer was obtained. FT-IR spectrum of SDFS (Figure 4) verified the structure of prepared monomer. The characteristic bands of sodium sulfonate groups were observed at 1083 and 1036 cm^−1^. In the ^1^H-NMR spectrum, all the related peaks were assigned (Figure 5) that confirming the structure and also the purity of SDFS as well. In the mass spectrum, presence of the parent ion at 467 Daltons was related to subtraction of sodium ion (23 Daltons) from the molecular ion (490 Dalton) that further confirmed the structure of monomer.

### Polymer characterization

**Scheme 3.**
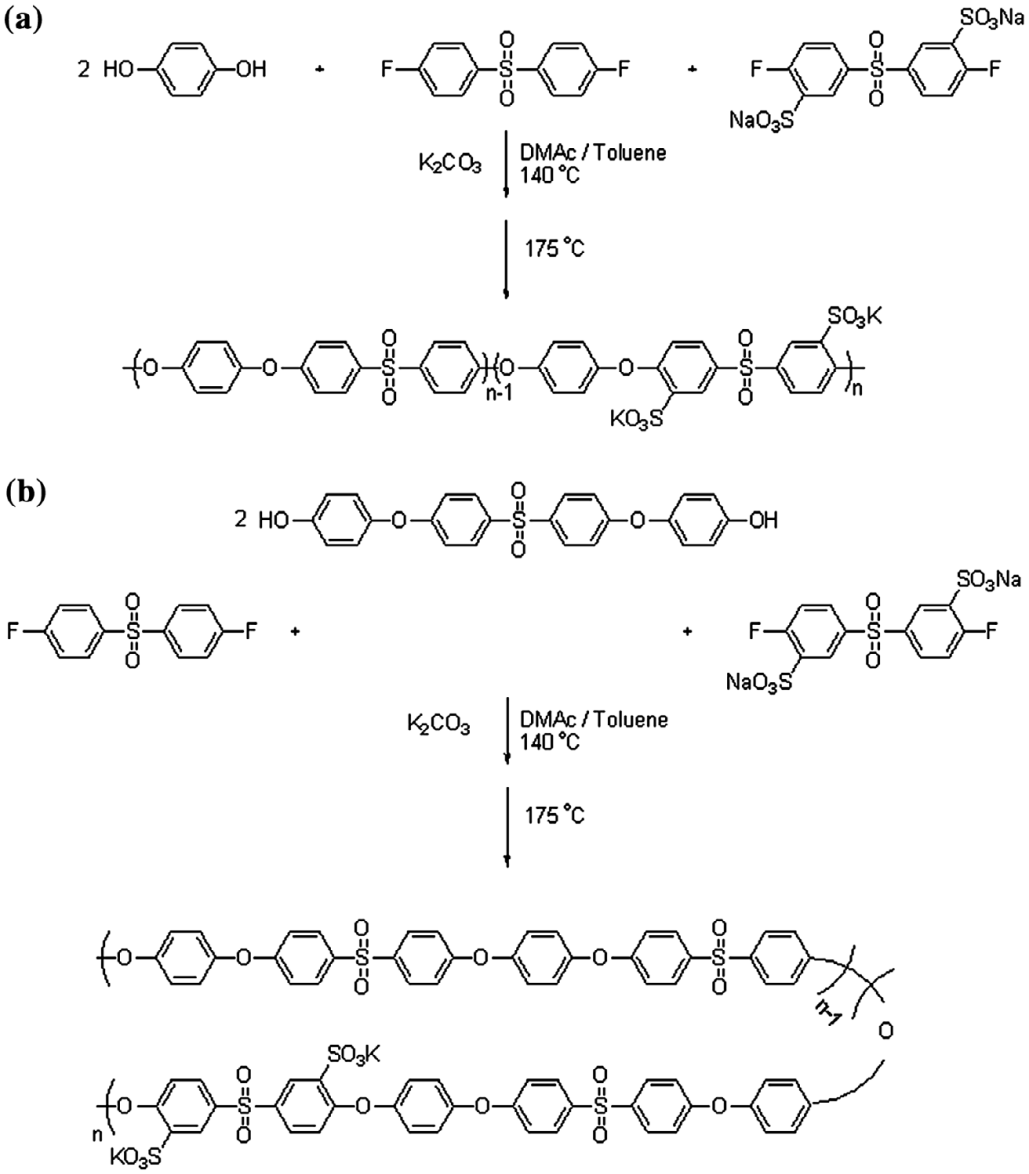
Synthesis of: (a) hydroquinone, and (b) SPHD-based poly(ether sulfone).

Two series of novel poly(arylene ether sulfone)s with different degrees of sulfonation (40, 50 and 60%) were prepared using direct copolymerization of 3,3′-disulfonated-4,4′-difluorodiphenyl sulfone (as sulfonated dihalide) in companion with 4,4′-difluorodiphenyl sulfone (as nonsulfonated dihalide) with diols including hydroquinone or 4,4′-(4,4′sulfonylbis-(1,4-phenylene)bis(oxy)) diphenol (Scheme 3). In both groups of copolymers it was observed that after solution casting and heating for solvent removal, copolymers with a higher degree of sulfonation showed less tendency to convert to coherent dry film, and this problem was intensified when SPHD-based copolymers were tried. FT-IR and ^1^H-NMR spectroscopy of copolymers (50% sulfonation) were used for characterization of the prepared polymers (Figures 6 and 7). Important characteristic bands in the FT-IR spectra were illustrated in Figure 6 completely. Another important observation from FT-IR spectra was that with increasing degree of sulfonation, the absorption intensity of the sulfonic acid groups increased.

Figure 7 shows ^1^H-NMR spectra of SPES-HQ and SPES-SPHD with peak assignments. The spectra of the polymers verified the molecular structure. No remarkable changes in the ^1^H-NMR spectrum of SPES-HQ and SPES-SPHD polymers were observed due to the fact that they have the same repeating unit structure. ^1^H-NMR was applied for structural as well as compositional determination of each group of copolymers. The degree of sulfonation of the various sulfonated polymers was defined by comparison of integration of protons in the copolymer structure. The two protons next to the sulfonate group (at ~8.25 ppm) on the sulfonated dihalide monomer were well separated from the other aromatic protons of all the copolymers and they were assumed as reference peaks. The calculated degree of sulfonation for sulfonated poly(arylene ether sulfone)s, were shown in Table 1. The results were reasonable and within experimental error i.e. they were typically within ± 10% of the expected values.

**Table 1.** Degree of sulfonation, water uptake, and IEC titration of synthesized copolymers.

Polymer	Expected degree of sulfonation (%)	Calculated degree of sulfonation (%)	Water uptake (wt %)	IEC titration (meq/g)
PES-HQ	40	39.87	31.3	1.63
	50	48.09	40.4	1.97
	60	52.85	45.2	2.19
PES-SPHD	40	35.31	45.1	0.88
	50	45.83	56.3	1.13
	60	51.81	61.8	1.28

### Polymer properties

Solubility of the polymers was studied in different solvents. All the sulfonated polymers with different degrees of sulfonation were soluble in polar aprotic solvents such as DMAc, NMP, DMF, and DMSO but were insoluble in water. The good solubility of the copolymers led to easy membranes formation by solvent casting method. Improved solubility of the polymers could be related to the presence of flexible ether units in addition to sulfone and sulfonic acid groups.

The inherent viscosities of the polymers were listed in Table 2. The viscosity values of SPES-HQ and SPES-SPHD were in the range of 1.02–1.85 dL/g indicating that successful polymerizations were done and high molecular weight polymers were obtained. In addition, tough and processable membranes were prepared from these polymers, which also originated from their high molecular weights. It might be supposed that the sulfonated poly(arylene ether sulfone) copolymers with a higher degree of sulfonation make the copolymer appear more bulky and contribute to the observed high inherent viscosities. So, interaction of sulfonic acid groups with solvent had major effect on the increased viscosity of the polymers. Similar trends of increased inherent viscosities with increased ionic contents have been reported by several researchers for a number of polymer systems.[7–13]

**Table 2.** Viscosity, molecular weight, and 2nd virial coefficient of copolymer*.

Polymer	Degree of sulfonation	Inherent viscosity (dL/g)	*M*_w_ (kDa)	2nd virial coefficient (mL mol/g^2^)
PES-HQ	40	1.02	129.0	0.0014
	50	1.23	81.1	0.0218
	60	1.31	70.0	0.0275
PES-SPHD	40	1.45	94.3	0.0045
	50	1.72	52.5	0.0151
	60	1.85	57.8	0.0201

^*^ Refractive index increment was about 0.204 mL/g.

IEC and water uptake are two important properties of sulfonated polymers that related to amount of H^+^ ion in materials. Typically, the IEC is determined by means of titration. Table 1 lists the IEC values of the copolymer membranes measured by a titration method as explained previously. In both groups of copolymers by increasing of the sulfonic acid content, the ion exchange capacity was increased. It was expected that in SPHD based copolymer larger hydrophobic/hydrophilic segments causing more free volume in copolymer structure and access to sulfonic acid groups become easier, however based on ^1^H-NMR spectroscopy data, the degree of sulfonation in PES-SPHD was lower than similar PES-HQ structures and the result was that the amounts of IEC in PES-SPHD structures were lower.

Water uptake of sulfonated polymers is known to have a great effect on membrane conductivity and mechanical properties. The obtained results for the water uptake of the prepared polymers showed the similar trend, this property in two groups of copolymer was increased as the degree of sulfonation increased that indicating more phase separation was occurred. The water uptake of both copolymers with 40 wt% degree of sulfonation was in the range of Nafion water uptake i.e. about 30 wt% (Table 1).

For membrane application the growth of molecular weights in these polymers is vital, so in order to have the estimation of molecular weight of polymers more sensible, laser light scattering (LLS) was applied. This technique uses for characterization of the molecules in solution, from which we can evaluate the molecular weight (*M*_W_) and the 2nd virial coefficient (A_2_).[48,49] The 2nd virial coefficient (*A*_2_) is a property indicating the interaction strength between the particles and the solvent. Dry dimethylacetamide (DMAc) was used as a good solvent (analytical grade) for the prepared copolymer without further purification. Four concentrations ranged from 0.1 to 0.4 mg/mL were prepared by dilution. After solution preparation, all copolymer solutions were filtered in order to remove dust and multi-chain aggregates. Malvern zetasizer laser light scattering was used with a He–Ne laser 633 nm, Max 4 mV as light source. The molecular weights were determined by applying the Rayleigh equation and all the measurements carried out at 25 °C. Based on Rayleigh equation for molecular weight determination by LLS instrument, the refractive index increment and absorption for each sample should be determined. All data was gathered in the laboratory by refractometer and collected in Table 2.

The LLS measures the intensity of scattered light (K/CR) of various concentrations (C) of solution samples at a single angle. The plot of this is called a Debye. The Molecular weight (*M*_w_) was determined from the intercept point on the *X* axis. i.e. K/CR = 1/*M*_W_ in Daltons. The 2nd virial coefficient (*A*_2_) was determined from the gradient of the Debye plot (Figure 8).[48,49] *M*_w_ and *A*_2_ for each copolymer sample were shown in Table 2. As expected, for higher degree of sulfonation, *A*_2_ was increased and for samples with *A*_2_ > 0, the copolymers behaved ‘like’ the solvent (DMAc) more than itself, and tend to stay as a stable solution. Additionally, it should be mention that *A*_2_ had reversed relation with the molecular weight. Another result was that no logical pattern was observed for the growth of molecular weight and degree of sulfonation.

Two other data can be extracted by LLS technique are zeta potential and conductivity. Zeta potential is a measure of the magnitude of the electrostatic repulsion/attraction between particles and is one of the known fundamental parameters to affect stability. In samples diluted with DMAc solvent, as the degree of sulfonation increased, zeta potential and conductivity of each sample were increased that indicating more separation of H^+^ ion from sulfonated polymers and getting more stable solution. In Table 3 zeta potential and conductivity of each sample was shown. According to the obtained results, zeta potential was decreased by increasing ionic strength due to the increasing concentration of macro ions and also chain overlap. This was an important conclusion for application of these polymers as fuel cell membranes due to the fact that proton transfer is an essential character for the performance of membrane.

**Table 3.** Zeta potential and conductivity of solutions.

Polymer	Degree of sulfonation (%)	Concentration (mg/mL)	Zeta potential (mv)	Conductivity (mS/cm)
PES-HQ	40	0.1	−31.0	0.0051
		0.4	−27.4	0.0136
	50	0.1	−33.2	0.0085
		0.4	−35.0	0.0169
	60	0.1	−34.2	0.0064
		0.4	−26.2	0.0187
PES-SPHD	40	0.1	−36.3	0.0055
		0.4	−28.6	0.0121
	50	0.1	−40.5	0.0073
		0.4	−35.2	0.0178
	60	0.1	−44.3	0.0081
		0.4	−39.3	0.0189

Thermal properties of SPES-HQ and SPES-SPHD copolymers that are important factors in membrane applications were investigated by TGA and DSC techniques. From the TGA curves (Figure 9) it was observed that thermally stable copolymers were obtained.[7–13] The TGA curves of copolymers in air atmosphere exhibited typical three major step degradation patterns. The initial degradation step was observed at room temperature to about 200 °C, could be attributed to the loss of water molecules absorbed by SO_3_H groups. The second main weight loss step started above 240 °C was assigned to the thermal degradation of sulfonic acid groups, while the third important step was above 500 °C could be related to the decomposition of the main polymer chain. Temperatures for 5% weight loss were summarized in Table 4 and as shown in Figure 9 (for 50% sulfonation) it decreased with increasing the degree of sulfonation. It was due to the fact that sulfonic acid groups degrade in lower temperature in comparison to copolymer main chain. On the other hand, SPES-SPHD copolymers were more stable and all three major decomposition steps occurred at higher temperature compared to SPES-HQ copolymers (Table 4 and Figure 9).

**Table 4.** Glass transition and decomposition temperatures of synthesized copolymers.

Polymer	Degree of sulfonation (%)	*T*_d 5%_ (°C)	*T*_g_ (°C)
PES-HQ	40	251.1	208.6
	50	244.7	230.3
	60	220.4	238.4
PES-SPHD	40	270.6	240.9
	50	253.5	246.4
	60	238.1	252.2

The copolymers showed an increase in the glass transition temperature (*T*_g_) with increasing degree of sulfonation (Table 4 and Figure 10). This was in accordance with the observed behavior in the ionomeric polymers due to the strong interaction between sulfonic acid groups. In general, SPES-SPHD copolymers had higher glass transition temperature vs. SPES-HQ copolymers because of the presence of more hard segments in SPES-SPHD series, needing more thermal energy for segmental movements.

## Conclusion

Two novel series of sulfonated poly(arylene ether sulfone)s were prepared and characterized. The effect of similar repeating unit with different sizes on the final properties of these polymers for application in fuel cell membrane was studied and compared. Investigation of their properties including molecular weight, zeta potential, conductivity, thermal behavior, and thermal stability revealed their potential application as fuel cell membrane. Comparison of these copolymers showed that polymers with larger size of repeating unit (SPES-SPHD) had superior properties for application as fuel cell membrane.

## Disclosure statement

No potential conflict of interest was reported by the authors.
